# Going beyond the disability-based morbidity definition in the compression of morbidity framework

**DOI:** 10.3402/gha.v7.24766

**Published:** 2014-09-24

**Authors:** Hiram Beltrán-Sánchez, Fahad Razak, S. V. Subramanian

**Affiliations:** 1 Center for Demography of Health and Aging, University of Wisconsin-Madison, Madison, WI, USA; 2 St Michael’s Hospital, University of Toronto, Toronto, Canada; 3 Li Ka Shing Knowledge Institute, Ontario, Canada; 4 Harvard Center for Population and Development Studies, Cambridge, MA, USA; 5 Department of Social and Behavioral Sciences, School of Public Health, Harvard University, Boston, MA, USA

**Keywords:** disability, morbidity, compression of morbidity, aging

## Abstract

**Background:**

As originally proposed by Fries, conceptualizing morbidity solely through associated functional limitation/disability (FL/D) remains the most widely accepted metric to assess whether increases in longevity have been accompanied by a compression of morbidity.

**Objective:**

To propose a departure from a highly restrictive FL/D-based definition of “morbidity” to a broader view that considers the burden of chronic diseases even when no overt FL/D occur.

**Design:**

We outline three reasons why the current framework of compression of morbidity should be broadened to also consider morbidity to be present even when there are no overtly measurable FL/D. We discuss various scenarios of morbidity compression and morbidity expansion under this broader rubric of morbidity.

**Conclusion:**

The rationale to go beyond a purely FL/D-based definition of morbidity includes: (1) substantial damage from chronic disease that can develop prior to overt FL/D symptoms occurring; (2) multiple costs to the individual and society that extend beyond FL/D, including medication costs, health care visits, and opportunity costs of lifelong treatment; and (3) psychosocial and stress burden of being labeled as diseased and the consequence for overall well-being. Adopting this broader definition of morbidity suggests that increases in longevity have been possibly accompanied by an expansion of morbidity, in contrast to Fries’ original hypothesis that morbidity onset (based on only FL/D) would be delayed to a greater extent than increases in survival. There is an urgent need for better data and more research to document morbidity onset and its link with increases in longevity and assess the important question on whether populations while living longer are also healthier.

In 1980, James Fries proposed the concept of *compression of morbidity*, which he defined as, ‘The amount of *[sic]* disability can decrease as morbidity is compressed into the shorter span between the increasing age at onset of disability and the fixed occurrence of death … Postponement of chronic illness thus results in rectangularization not only of the mortality curve but also of the morbidity curve’ (1, p. 133). In Fries’ view, morbidity was represented by disability which resulted from chronic illness: ‘Chronic illness now is responsible for more than 80 per cent of all deaths and for an even higher fraction of cases of total disability. Atherosclerosis (including coronary-artery disease and stroke), arthritis, adult onset of diabetes, chronic obstructive pulmonary disease (including emphysema), cancer, and cirrhosis represent the overwhelming majority of our health problems … Generally, they develop slowly and asymptomatically below a clinical threshold, at which the process becomes clinically evident, progresses, and often culminates in death or disability. Disability and lowered quality of life due to the most prevalent chronic diseases are thus unescapably linked with eventual mortality’ (p. 132). Put simply, Fries’ conception of morbidity required chronic illness to have overtly measurable functional limitation/disability (FL/D) in order to be considered relevant.

The majority of the assessments on whether additional years of life have been morbidity-free or not, consequently, have relied solely on measuring the onset (or levels) of FL/D over time to test whether there has been a compression or expansion of morbidity, as opposed to also considering the onset of the chronic disease themselves. There have been variants to this basic framework, such as the dynamic equilibrium, (2) which posits that the severity and progression of chronic disease would change at the same pace as mortality improvements so that the progression of disease would be stopped at early stages, resulting in potentially more disease in the population but disease with stable consequences in terms of FL/D. However, any subsequent modifications to the basic idea of Fries did not fundamentally alter the core tenet, that is, morbidities are restricted to be impairment of individuals’ ability to perform certain functions [e.g. measured through scales such as activities of daily living (ADL)] that could result as a consequence of chronic disease.

In this comment, we develop an argument to not restrict ‘morbidity’ simply to chronic illness with overtly measurable FL/D but also include chronic disease even if they do not lead to FL/D. We then summarize the various scenarios of ‘compression’ and ‘expansion’ under a broader rubric of morbidity definition. In what follows, we use the term ‘disability compression’ to refer to changes in FL/D indicators (incidence or prevalence) whereas ‘morbidity’ will be used to describe ***both*** disease and/or FL/D related to disease. For instance, if a person has diabetes but no FL/D, we would still consider them to have morbidity. If they have diabetes and FL/D, they have both morbidity and disability.

Before discussing morbidity compression more broadly, we first consider the evidence for disability compression. Whether disability compression has occurred is uncertain, in part due to available data, the FL/D measure employed, and the age range included in the different studies. For instance, in the US, some research suggests a small postponement in age at incidence from disability [ADL and instrumental activities of daily living (IADL)] between 1992 and 2003 for people aged 65 for severe (people unable to complete at least three ADL’s) and moderate (disabled in one or two ADL’s) disability, with increasing rates of recovery from disability (3). However, a cross-survey comparison in five major studies in the US shows that ADL disability prevalence had no significant change from 1999/2000 to 2008 for people aged 65 or older; although, one survey (the Health and Retirement Study-HRS) shows a decline for people aged 85 or older in a 4-year period (2000–2004) while other survey (Medicare Current Beneficiary Survey-MCBS) suggests this decline had occurred for a longer period (2000–2008) (4). Meanwhile, among younger adults (aged 55–64) disability prevalence (ADL and IADL) has been shown to be increasing over 2000–2004 (4); a result that has also been reported for the rate of people needing help with personal care activities at ages 50–64 (5) and for ADL disability for ages 40–64 (6, 7). The latter results indicate that incidence of disability is likely occurring at earlier ages among younger adults; these are the same cohorts that are experiencing high prevalence of health risk factors such as obesity and dyslipidemia (8).

## A rationale for going beyond disability-based morbidity

Moving beyond a disability-based definition of morbidity (i.e. considering an individual to have morbidity even when they do not have FL/D) would entail considering the broader array of chronic disease. A chronic disease is a condition that can be controlled but not cured, including metabolic derangements such as type 2 diabetes and cognitive disorders such as Alzheimer’s disease. We define as disease those chronic conditions that are included in the International Classification of Diseases (ICD-10) (9) such as hypertension, diabetes, obesity, and mental and behavioral disorders (e.g. Alzheimer’s disease). These conditions not only increase the likelihood of developing FL/D but also raise medical costs, result in sub-clinical damage not measured through FL/D, and have associated psychosocial and stress related burdens. We outline three reasons why assessment on whether increases in life expectancies have been accompanied by a ‘compression’ or ‘expansion’ of morbidity should consider situations even when there is no overt FL/D.

First, substantial damage from chronic disease can develop prior to overt symptoms occurring. For instance, decline in kidney function from diabetes is largely asymptomatic until function is severely impaired. Diabetes, hypertension, and dyslipidemia all will substantially elevate risk of heart attack or stroke but the person may be ‘symptom free’ until the actual event (e.g. heart attack) occurs. The concept of ‘silent risk’ is captured in risk scores, such as the Framingham Risk Score, that recognize that even though no FL/D are occurring, the presence of conditions such as dyslipidemia or hypertension are associated with considerable underlying physiologic damage (10).

Second, diseases are accompanied by multiple costs to the individual and society that extend beyond FL/D, and these costs may be expanding with earlier time of diagnosis and longer duration of illness. Evidence suggests that individuals with diseases such as diabetes are not being diagnosed at an older age (11), rather, they are experiencing a delay in the progression of the disease where good control can be achieved through medical therapy [which is consistent with the dynamic equilibrium theory (2) and with the fourth stage of the epidemiologic transition: ‘the age of delayed degenerative diseases’ (12)]. But control of diseases such as diabetes and hypertension are associated with medication costs, health care visits, and opportunity costs, and most of these conditions require lifelong treatment. For example, in diabetic patients there is evidence that achieving clinical treatment targets (a lower value of HbA1c) may be associated with increasing patient burden to the point of net harm, especially among older adults (13). This reflects a potential trade-off whereby improvement in clinical outcomes (and reduction of some forms of diabetes related comorbidity) may actually worsen overall quality of life.

Finally, diseases are also accompanied by social stigma/psychosocial burden and stress of being labeled as diseased. For example, in patients with a diagnosis of diabetes, two major psychosocial themes that emerge are anxiety and fear around complications of diabetes with associated hopeless and depression, and work-related discrimination and public misunderstanding around diabetes (14). There is also evidence suggesting that psychosocial burden and stress over long periods of time can exacerbate disease consequences, the so-called allostatic load (15, 16).

## Alternative scenarios of morbidity compression and expansion

**Fig. 1:**
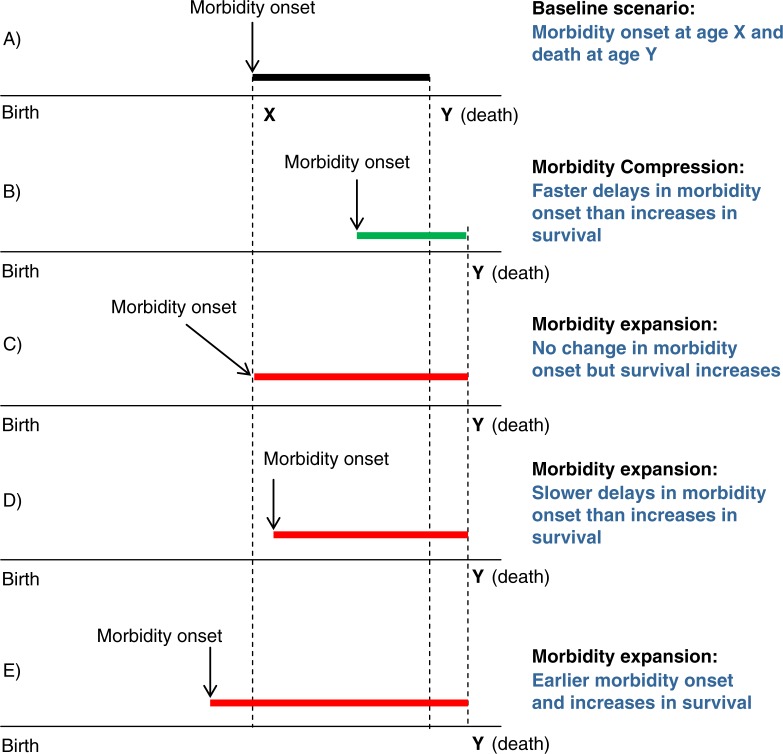
Models of compression/expansion of morbidity.

We present different scenarios that could result from the interplay of age of onset of morbidity (using the term to comprehensively refer to both disease and/or FL/D related to disease) and the increase in survival at older ages Fig. 1. Examples are given relative to Fig. 1(A), a baseline case in which morbidity onset occurs at age X and death occurs at age Y. Figure 1(B) shows the scenario posited by Fries whereby morbidity onset is delayed to a greater extent than increases in survival. However, three other scenarios all suggesting expansions of morbidity are equally plausible: survival increases (i.e. increasing average age at death) that are not accompanied by delays in age at onset of morbidity (C), lesser delay in age at onset of morbidity X relative to age at death Y (D), and onset of morbidity occurring at earlier ages while survival continues to increase (E).

We posit that scenario illustrated in Fig. 1(E) is highly plausible. Although direct evidence on age at diagnosis from nationally representative cohorts is not available, indirect evidence on the health status of the adult US population suggests morbidity expansion when the concept of morbidity includes the broader array of chronic disease. For instance, the age-adjusted incidence of diagnosed diabetes more than doubled between 1995 and 2010 among people aged 18–74 (11) and there was an increase in the prevalence of adults who reported having had a stroke or cancer between 1998 and 2006 (17). Similarly, the prevalence of disease such as hypertension in the adult US population appears to be on the rise. For example, recent evidence from the National Health and Nutrition Examination Survey (NHANES) indicates that one-fifth of the adult population in 2010 (aged 20 +) has at least three of the following: high glucose, high triglycerides, low HDL-cholesterol, high waist circumference, or high blood pressure (18); the prevalence of overweight and obesity showed a continuous increase in the 1990s among adults (aged 20–74) with recent increases in abdominal obesity (18), and the age-standardized prevalence of hypertension increased between 1988–1994 and 2007–2010 among people older than 20 years (11).

Among older adults (aged 65 or older) in the US, there is also evidence of a rise in the incidence of major chronic disease based on reconstructions of individual’s medical history from Medicare data (19). While there was a decline in the incidence of some cardiovascular events (e.g. angina pectoris and stroke) and some cancers (e.g. colon and prostate) between 1992 and 2005, there was also a rise in the incidence of diabetes, renal disease, lung cancer, and Alzheimer’s disease.

These patterns have also been accompanied by intensive use of medications. For example, the use of anti-hypertensive and lipid modifying agents in the adult US population significantly increased in the last decade (18). These important sources of morbidity currently afflicting the population are likely to lead to a potential scenario whereby individuals would live longer – in part due to improvements in case fatality rates from conditions such as cardiovascular disease (CVD) as well as primary prevention of CVD within individuals with diseases such as hypertension and dyslipidemia – but with a higher morbidity burden.

## Compression versus expansion of morbidity: future research

In this comment, we present a rationale for going beyond the FL/D view of morbidity to incorporate a broader view that considers sub-clinical damage, psychosocial and stress burden, and increased cost associated with disease. This broader view of morbidity simply brings back the basic definition of this concept. As the epidemiologic profile of populations change over time and our understanding and ascertainment procedures about pathology improve, there should also be a change in how we conceive and measure morbidity. More than three decades have passed since Fries proposed the framework of compression of morbidity and advocated for FL/D as markers of morbidity; yet, these indicators still dominate the assessment of this framework. Although indirect evidence suggests morbidity expansion rather than compression (as presented above), there is an urgent need for systematic research to document morbidity patterns of major chronic disease and their link with increasing survival at older ages.

Many recent health surveys collect data on chronic disease and underlying biomarkers of health allowing for rich models to understand changes in morbidity patterns and their link with mortality and survival at older ages (8, 20). Assessing whether compression of morbidity is truly occurring would require representative data on incidence of major chronic disease. Research on FL/D, for example, has already pursued this endeavor by using comparable FL/D measures with micro-simulation methods to infer incidence and recovery from disability from nationally representative surveys (either cross-sectional or longitudinal) (3, 21). Although some high-income countries (e.g. OECD members) have survey data on FL/D to assess disability compression, there is no data to assess disease onset of major chronic disease in these countries.

In epidemiological and clinical research, there is no nationally representative dataset in the US that allows one to assess incidence of major chronic disease. For example, major studies of cancer [e.g. the Surveillance, Epidemiology, and End Results (SEER) nine registry database] and heart disease (Framingham Heart Study) keep records of incident events with consistent diagnostic criteria for meaningful time trend analyses but are not nationally representative. National health surveys (e.g. NHANES) are cross-sectional in nature, limiting our understanding of disease onset as these conditions slowly develop over the individual’s life course and are often undiagnosed. Nonetheless, major strides have been made in recent years to overcome these data limitations by reconstructing, under certain assumptions, individual’s medical history from Medicare records to assess disease onset (19, 22, 23). Operationalization of the broader framework of morbidity that we propose will be challenging. Although diagnosis of diseases such as diabetes, hypertension or dyslipidemia is straightforward and well-established criteria exist for each condition, capturing and quantifying elements related to sub-clinical damage, psychosocial burden and cost is more challenging. This remains an important area for future research.

### Discordance between mortality projections and disease burden

A broader view of morbidity that considers the full impact of chronic disease could have important implications for public health policies. For instance, typical demographic and actuarial projections of mortality and life expectancy rely on extrapolations of past trends in demographic indicators with no attention paid to disease epidemiology and how chronic disease is expanding over time (24). Recent forecasting methods indicate that considering known health risk factors such as smoking and obesity will lead the US Social Security Administration Trust Fund to run out 2 years earlier than the government has predicted (24, 25). A broader morbidity measure that includes chronic disease would suggest the US Medicare Trust Fund would be exhausted even earlier as medical costs associated with lifelong treatments are likely to grow in the future.

An expanded view of morbidity has important implications to individuals, societies and governments. Chronic diseases are not only accompanied by economic costs to the individual resulting from earlier time of diagnosis and lifelong treatments but they are also associated with detrimental quality of life as a result of psychosocial and stress burden. Major improvements in adult mortality in the past decades will inevitably unfold as population aging in the years to come with an increasing number of people reaching older ages in most developed countries. It is imperative to assess the health status of this growing population. Societies and governments could face many challenges if morbidity is expanding as age patterns of public transfers typically favor older adults suggesting that national accounts would become even more unbalanced.

In summary, assessing compression of morbidity requires a broader view on morbidity than just FL/D-based indicators. If we want to assess the true impact of chronic disease, we must consider impact across domains related to FL/D, cost and psychosocial burden. This broader conception is a better representation of the burden and cost of illness to individuals and society and will allow for improved estimates of the impact of the disease on health care expenditure. This conception is an effort to more realistically capture the impact of chronic disease when assessing how a population is doing in terms of health. More research needs to be done in this area with particular emphasis on evaluating the implications of a broader view on morbidity for health care policies and population projections. Such detailed cost-benefit assessments accompanied by a broader view on morbidity need to be part of the discussion on compression of morbidity.
